# SOI MEMS Electro-Thermal Actuators for Biomedical Applications: Operation in 0.9% NaCl Solution

**DOI:** 10.3390/mi15070881

**Published:** 2024-07-04

**Authors:** Thomas Sciberras, Ivan Grech, Marija Demicoli, Bertram Mallia, Nicholas Sammut, Pierluigi Mollicone

**Affiliations:** 1 Department of Mechanical Engineering, Faculty of Engineering, University of Malta, MSD 2080 Msida, Malta; pierluigi.mollicone@um.edu.mt; 2 Department of Microelectronics and Nanoelectronics, Faculty of Information and Communications Technology, University of Malta, MSD 2080 Msida, Malta; ivan.grech@um.edu.mt (I.G.); nicholas.sammut@um.edu.mt (N.S.); 3 Institute for Sustainable Energy, University of Malta, MXK 1531 Marsaxlokk, Malta; marija.demicoli@um.edu.mt; 4 Department of Metallurgy and Materials Engineering, Faculty of Engineering, University of Malta, MSD 2080 Msida, Malta; bertram.mallia@um.edu.mt

**Keywords:** microelectromechanical systems (MEMS), numerical modelling, electro-thermal actuator (ETA), V-shaped driver, fluid–structure interaction, finite element analysis, submerged, underwater, aqueous, biomedical

## Abstract

In recent years, the immense potential for MEMS devices in the biomedical industry has been understood. It has been determined that, among their many plausible functions, their use may also extend to single human red blood cell diagnostics, whereby biomarkers of quantifiable magnitudes may be detected. Without a doubt, the mechanical and thermal specifications by which potential devices must be able to function are very strict. Among them is the ability to operate while fully submerged in aqueous solutions. In this work, six devices were modelled numerically in deionised (DI) water and 0.9 wt% NaCl solution, the results of which were validated experimentally. The mechanical performance of the different devices when fully submerged in 0.9 wt% NaCl solution is hereby discussed. With the exception of one, all the devices in their current configuration are confirmed to be suitable candidates for biomedical applications.

## 1. Introduction

Recent studies have determined that certain medical conditions induce detectable traces, often termed biomarkers [1], on human red blood cells (RBCs) [2]. Two such conditions include sickle cell anaemia [3,4] and diabetes [5,6,7]. Recent advances in the miniaturisation of devices have enabled innovation in modern medicine and surgery in that single-cell measurements are now possible for diagnostics [8]. While commonly available diagnostic methodologies such as ektacytometry have, to date, been the industry standard [5,9], they make use of multiple cells in a solution and, therefore, test a heterogenous sample of cells having a variety of properties [8]. Postprocessing routines are, hence, often performed on averaged values of the test sample. Any potential doubts associated with errors brought about by the testing of heterogenous samples are automatically eliminated with the use of ex vivo single-cell measurement methods [8]. While there exist other single-cell methods such as atom force microscopy (AFM) [6] and optical tweezers [10], MEMS are often still the preferred candidates. AFM entails high-end setups which can be expensive and require highly trained personnel to perform the procedure. Also, when attempting to characterise specimens such as a single RBC in a solution, one must first affix the specimen to a fixed surface with the use of specialised chemicals such as poly-L-lysine [11]. This may affect the RBC response to the scanning process. Single-cell characterisation via optical tweezers is a novel methodology that often requires the attachment of glass microbeads to the cell. The beads are then used to impart a tensile strain on the RBC with the use of highly focused laser beams. Dedicated equipment along with skilled personnel are also required to perform such procedures. MEMS fabrication processes are well established and readily available. Moreover, the cells do not require the attachment of or to tertiary objects. Also, it is anticipated that no highly specialised skill shall be required to operate MEMS devices. MEMS devices, therefore, pose themselves as potential tools within this niche biomedical sector of biophysical property characterisation activities [12,13,14,15,16,17,18].

As detailed in [15], for a MEMS device to qualify as a potential human RBC manipulation and/or characterisation tool, there is a group of specifications by which the candidate device must comply. These include the following:

- Thermal Criteria: As shall be seen later in this paper, MEMS electro-thermal actuators (ETAs) may be required to operate whilst fully submerged in aqueous media. For this reason, an elementary specification is that the device must not generate temperatures higher than the boiling temperature of the aqueous medium. In the current work, a maximum temperature of 100 °C was set as the temperature specification, as also implemented in [19]. This allows for bubble-free procedures that, in turn, allow for more accurate optical measurements. Secondly, human RBCs have a predefined temperature limit within which they maintain their original integrity. A previous work by Singh et al. [20] deduced that the decrease in deformability of a RBC is “highly significant” at test temperatures below 21.5 °C. In another publication by Xu et al. [21], the authors recalled that, below 22 °C, phosphatidylcholine lipids transition from a liquid-like state to a near-crystalline configuration. This implies that RBC stiffness increases at temperatures below 22 °C, therefore confirming the observations by Singh et al. [20]. Moreover, in [21], it was observed that stiffness alterations in RBCs in whole-blood when tested between 23 °C and 37 °C were negligible. More recent studies by Marai et al. [22] determined that human RBC stiffness in whole-blood samples was barely altered when tested between 37 °C and 39 °C. At temperatures above 40 °C, however, RBC stiffness begins to decay gradually in whole-blood samples. When human RBCs are suspended in aqueous saline solutions (phosphate-buffered saline, PBS) rather than whole-blood, the rate of stiffness decay is even more exaggerated. The target fluid for cell manipulation in this study was identified as a 0.9 wt% NaCl solution rather than whole-blood. Considering the above criteria, the temperature range for cell manipulation and characterisation in this work was defined as no less than 22 °C and no more than 40 °C [20,23,24]. This requirement implies that the designated characterisation or manipulation region on the device (specifically the end-effector tips) must not experience temperature increases beyond these set limits [15].
- Physical Attributes: A device must be able to physically compress a human RBC by at least 7 µm along its largest diameter. This is because human RBCs are typically characterised by their major diameter, in the order of 10 µm, and must travel through capillaries with a 3 µm diameter [25].
- Operational Media: In order for a human RBC to maintain its original integrity, it must be kept in an isotonic fluid [26,27]. A 0.9% NaCl solution is considered an isotonic solution [28]. Given the micron-size footprint of MEMS devices, partial immersion may be a challenge in itself. In the current work, it was specified that the device must be able to operate in fully submerged conditions. In such conditions, a number of issues may arise. Among them are electrolysis [29] and aqueous corrosion [29]. Similarly to why boiling ought to be avoided, the electrolysis of a fluid must also be avoided since gas evolution may impede displacement measurements and also affect corrosion kinetics. The authors of [30] proposed using high-frequency AC pulses with a mean voltage of 0 V to avoid the occurrence of electrolysis and corrosion phenomena. This strategy was successfully implemented in [15,19], and a similar methodology was adopted in our study.

To date, work available in the literature concerning MEMS electro-thermal actuators (ETAs) intended for biomedical applications and satisfying the above criteria is still somewhat limited. The current work aimed to present the performance of MEMS ETA devices fully submerged in a 0.9% NaCl solution and shed light on their suitability for human RBC manipulation. Furthermore, this work was validated by characterisation procedures carried out in submerged conditions. Device performance in a 0.9% NaCl solution was compared to that in air and DI water previously reported in [19,31], respectively. 

The main focus of this work was the electro-thermo-mechanical performance of devices whose geometrical configurations and working principles had been presented in previous works [17,29]. Structural configurations and material properties were carried over into this work without changes. All the ETAs presented hereunder incorporated a cascaded V-shaped driver that was used to produce the output displacements at their end-effectors. Device configurations and newly assigned designations shall be described in the subsequent sections. Note that, although the devices’ geometry was presented in previous publications, their functional performance in a saline solution had not yet been assessed and reported.

## 2. Materials and Fabrication Process Overview

**Figure 1:**
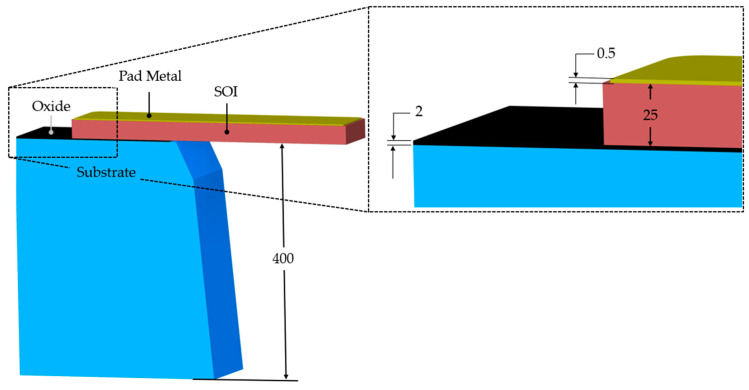
Typical SOIMUMP stack (not to scale). Image generated based upon the fabrication procedures described in [32]. Dimensions are in µm.

The MEMS ETAs analysed in this work were fabricated using the Silicon-on-Insulator Multi-User MEMS Processes (SOIMUMPs™) [32]. This commercially available process is a simple, three-mask process and comes with an n-type doped single-crystal silicon (SCS) as the SOI layer. With reference to Figure 1, the SOI layer has a thickness of 25 µm, which implies good out-of-plane stiffness characteristics. The process allows for a pad metal layer consisting of a 0.5 µm gold layer over a 0.02 µm thick layer of chromium. Note that the chromium layer is not shown in Figure 1. This pad metal layer may be used for electrical routing as well as the external electrical power source interface’s location for the device in question. Beneath the SOI layer is a 400 µm thick substrate material, which is a high-resistance silicon. In the subsequent sections concerning numerical modelling, the resistivity of the substrate material was assumed to be 50,000 Ω·µm [15,32]. Separating the SOI and the substrate is a 2 µm thick silicon oxide layer, electrically isolating the pair [32].

The thermophysical properties of the SOI and pad metal layers as assumed in this work are given in Table 1. Note that the coefficient of thermal expansion (CTE) of the SOI layer was assumed to vary with temperature [32]. The CTE–temperature relationship used in this work may be seen in Figure 2 of [15] and Figure 4 of [31]. 

It is worth noting that a low-resistivity SOI was opted for, since this lower resistance, compared to that of aqueous media, allows for the electrical current to favourably flow through the semiconductor rather than the fluid. Note that the electrical resistivity of DI water and 0.9 wt% NaCl are 2.4 × 10^9^ µΩm [19] and 6.21 × 10^5^ µΩm, respectively, at 22 °C. Also, the SOI layer is the electro-thermo-mechanical foundation of the compliant mechanisms presented here, in that it acts as both the electrical routing of the devices as well as the structural mechanism which actuates via Joule heating brought about by the current flow. The device’s designations are shown in Table 2.

Devices 1 to 4 were inspired by the ETA developed by Qu et al. [11], who developed a V-shaped driver combined with a beam-type amplifier. Here, however, the V-shaped driver was replaced with a cascaded V-shaped driver to actuate a beam-type amplification mechanism. Devices 1 and 2 incorporated a curved-type pivoting hinge inspired by the flexure hinge used by Yallew et al. [14], whereas devices 3 and 4 had a linear-type clamped pivoting beam. Device 5 was a tweezer-type ETA, initially inspired by the operational characteristics of a typical U-shaped ETA developed by Cauchi et al. [17,18]. The design was then extended to include other tweezer-type MEMS such as those developed by Vurchio et al. [19] and Yallew et al. [14]. Device 6 had a ram-type end-effector connected to the cascaded V-shaped driver described above. Device 6 was inspired by the works of Zhang et al. [15] and Barazani et al. [16], who implement a compression-type end-effector coupled directly to a single V-shaped ETA and a cascaded V-shaped ETA, respectively. In this work, the ram, which is an integral part of the device’s tertiary amplification mechanism (or end-effector), makes this device particularly attractive for compression-type procedures such as the characterisation of cells or micro-objects.

## 3. Numerical Modelling

Numerical models for operation in air and DI water were previously reported in [19,31], respectively. In both cases, a sequentially coupled method was used for the electro-thermo-mechanical performance prediction of the devices in their respective media. Similarly, in this work, a sequentially coupled method, as described in [19], was implemented in Ansys^®^ Academic Research Mechanical, Release 21.1, to virtually characterise the devices’ electro-thermo-mechanical performance in a 0.9% NaCl solution. The thermophysical properties of the aqueous solution used in the analyses of the current work are shown in Table 3. Note that the electrical resistivity of the aqueous solution was measured rather than extracted from the literature. 

To recap, since conduction to the surrounding medium has been postulated to be the predominant mode of heat transfer in MEMS ETA devices [33], an upstream electro-thermal analysis was set up with the device as well as the surrounding medium, all modelled in a purely Lagrangian framework. Convection and radiation phenomena were ignored, and heat loss by the device to the surrounding medium was, thus, only possible via conduction. Here, all the geometries were discretised into higher-order, 10-node tetrahedral finite elements with voltage and temperature degrees of freedom. Within this module, Joule heating resulting from an electrical current was calculated, and spatially varying temperatures could be used for subsequent structural analyses. 

**Table 3:** Material properties of the 0.9% NaCl solution, as utilised in the numerical models. Note that the properties are at a temperature of 22 °C.

Property	Value
Thermal conductivity [34], k (W/m.K)	0.594
Specific heat capacity [35], c (J/kg.K)	4182
Electrical resistivity (Ω.m)	0.621
Density [36], (g/(cm)^3^)	0.1004

**Figure 2:**
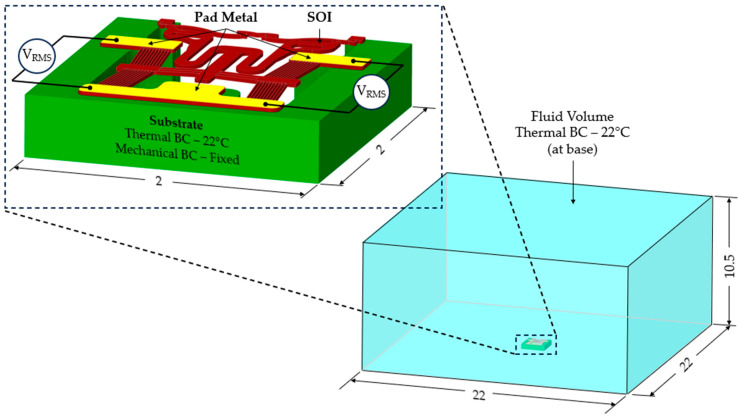
Computer-aided design (CAD) models of numerical model environments. Electro-thermo-mechanical loads and BCs are included. (**Top left**): MEMS device domain; (**bottom right**): fluid volume domain. The device represented in the figure is device 6. The major dimensions, loads, and BCs illustrated are applicable to all devices. Note that the dimensions are in “mm”. With reference to the device domain (**top left**), a potential is applied across the anchors. The potential is applied from 0 to 5 V in steps of 1 V. The substrate temperature is fixed at 22 °C in the upstream module. The substrate base is mechanically clamped in the following downstream module. With reference to the fluid volume, the fluid base is thermally clamped at 22 °C in the upstream module.

The spatially varying temperatures developed in this device were then sequentially transferred to a downstream module where only the MEMS constituents were present. The MEMS ETA’s geometry was discretised with higher-order 10-node tetrahedral elements having displacement degrees of freedom. The volume representing the fluid domain was removed for analysis in this downstream structural module. Here, thermal strains as a function of the temperature calculated in the upstream module were calculated, and, therefore, end-effector tip displacements could be characterised numerically. Figure 2 includes a graphical representation of such a sequentially coupled numerical model, including loads and boundary conditions (BCs). Note that steady-state solvers were used in both the electro-thermal and structural modules.

## 4. Experimental Testing

The electro-thermo-mechanical performance of the MEMS ETAs was characterised experimentally using MEMS-dedicated testing equipment—Cascade Microtech Summit 11K/12K B-series station (Cascade Microtech Inc., Beaverton, OR, USA)—by measuring the tip displacements. Tip displacement measurements were possible with the station’s inbuilt microscopy system. Three-axis micro-positioners were used to supply an electrical potential difference across the device anchors through 10 µm diameter voltage probes. Five separate displacement measurements were taken per input voltage. This served two purposes: ensuring measurement repeatability and defining the measurement error. The measurement error was defined as the standard deviation of the five readings. At present, there is no tool available at the authors’ disposal to accurately measure out-of-plane deformation. Despite this, out-of-plane distortion was deemed negligible since, at no stage during the testing, was there a loss of focus observed at the device tips. As shall be elaborated upon hereunder, testing under the premise of this work was performed only in the 0.9% NaCl solution. Testing in air and DI water had been performed in previously published work.

### 4.1. Frequency Independence Study

Similar to the experimental procedures adopted in [15], a frequency independence study was adopted in this work. With reference to [15,29], the scope of this exercise was to verify that the mechanical response of the devices at a given root mean square (RMS) voltage was similar to that exhibited when powered using a direct-current (DC) voltage of the same value [15]. A 15 kHz sine waveform with a 0 V DC offset was used when testing in the 0.9% NaCl solution. The amplitude voltage was incremented from 0 V to 6 V in steps of 0.5 V. Prior to performing this exercise, a modal analysis was performed in Ansys^®^ Academic Research Mechanical, Release 21.1, to verify that no resonant modes were excited at 15 kHz.

### 4.2. Testing in Aqueous Solutions

**Figure 3:**
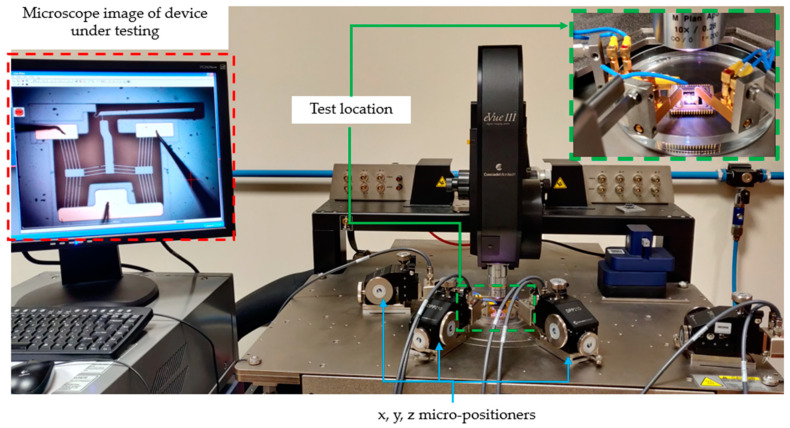
Typical experimental setup for testing in aqueous media.

Testing in aqueous media was also undertaken using the same procedure as that detailed in [15]. The devices were totally submerged in their respective solutions inside polymer Petri dishes. A typical test setup can be seen in Figure 3. A cleaning procedure developed in [15] was also implemented here and consisted of the following steps [15]:

- The devices were first soaked in high-purity isopropyl alcohol (IPA) for 10 min;
- The devices were then removed from the IPA and rinsed for three consecutive times in the aqueous medium in which they were to be tested;
- Finally, they were submerged in their respective medium, ready for testing.

## 5. Results and Discussion

The numerical and experimental results shall be presented in this section. The thermo-mechanical performance of the devices in the 0.9% NaCl solution shall be compared to that in DI water previously reported in [15,19]. Reference shall be made to the device specifications elaborated upon in Section 1, and individual device suitability for underwater biomedical procedures shall be discussed. 

### 5.1. Electro-Thermal Characterisation

The electro-thermal characterisation of the devices was only performed numerically due to the unavailability of the necessary experimental hardware. 

**Figure 4:**
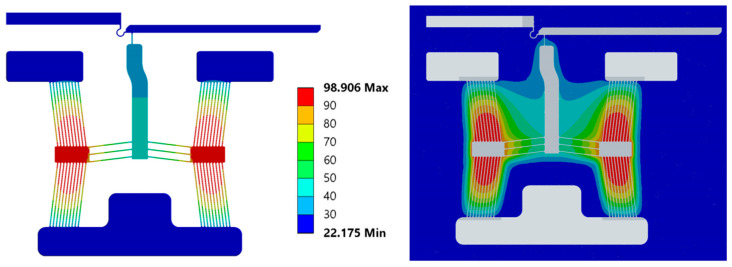
Numerical temperature contours in the device (**left**) and surrounding fluid (**right**) of device 1 at an input voltage of 5 V_RMS_ at steady-state operation. Temperature legend in °C.

A numerically derived electro-thermal characterisation exercise is a critical element in the design and development process of electro-thermal actuators for applications involving temperature-sensitive subjects such as the target application of this work, i.e., the manipulation and characterisation of human RBCs. This section shall reveal the suitability of devices 1 to 6 to adhering to the thermal specifications detailed in Section 1, according to which no point on the devices must reach the boiling point of the fluid and the device’s end-effectors may not exceed a temperature of 40 °C so as not to damage the test subject. Note that the maximum temperature is typically exhibited on the device’s primary apex, as shown in Figure 4.

**Figure 5:**
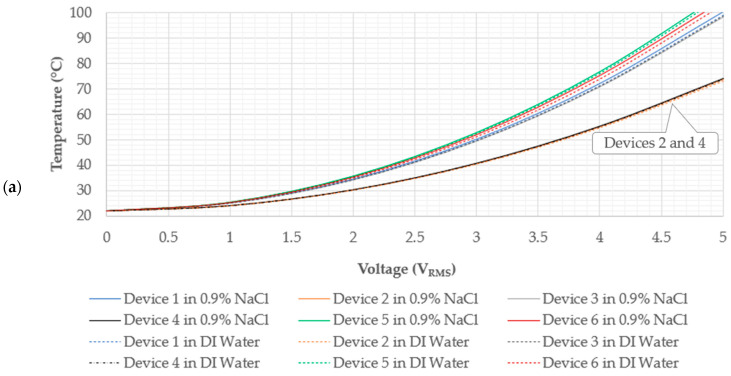

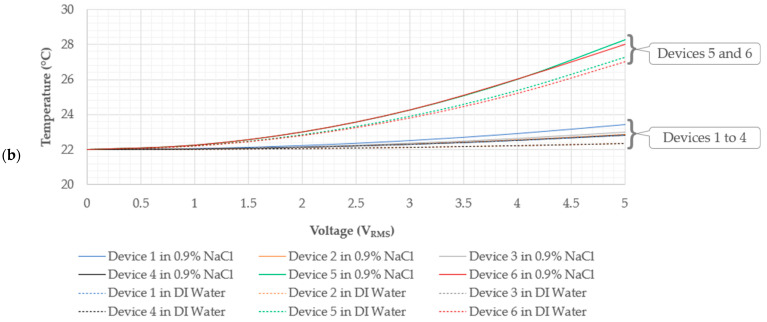
Steady-state temperature versus RMS voltage graphs as calculated numerically at the (**a**) device’s primary apex (maximum temperature) and (**b**) device’s tip. Temperature versus voltage data of devices 3, 5, and 6 in DI water have been reprinted with permission from [19] Sciberras, T.; Mollicone, P.; Demicoli, M.; Grech, I.; Sammut, N.; Mallia, B. “MEMS Electrothermal Actuators for Underwater Manipulation and Mechanical Characterisation of Human Red Blood Cells”, in *2023 Symposium on Design, Test, Integration & Packaging of MEMS/MOEMS (DTIP)*; 2023; pp 1–4.

Figure 5 includes the numerically derived temperature versus the RMS voltage graphs of the device’s maximum temperature (a) and the tip or end-effector temperature (b). With reference to Figure 5, it is evident that devices 2 and 4 adhere to the thermal specifications denoted in Section 1 up to a voltage of 5 V_RMS_. This is because device 2 exhibits maximum temperatures of 73.0 °C and 73.7 °C in DI water and saline, respectively, whereas device 4 exhibits maximum temperatures of 73.8 °C and 74.2 °C in DI water and saline, respectively, at a maximum applied voltage of 5 V_RMS_. Also, both devices do not exhibit temperatures higher than 23 °C at their tip at 5 V_RMS_. Note that, as explained in [15], one reason why devices 2 and 4 exhibit lower maximum temperatures when compared with the other devices is that they (devices 2 and 4) have only five beams per side of their shuttle, implying that they have a higher electrical resistance and, therefore, less Joule heating per applied volt. Another reason is that, in the case of devices 2 and 4, the beams are further apart, meaning that it is more difficult for the beams to contain thermal energy in their vicinity by conduction through the fluid. Recall that conduction is being mentioned here since the numerical models applied in this work are assuming that conduction is the only heat transfer mechanism possible. Note that, due to hardware limitations, voltage ranges beyond 5 V_RMS_ and 4.24 V_RMS_ are not permissible in the DI water and 0.9% NaCl solutions, respectively. Nevertheless, devices 2 and 4 would have safe operating voltages up to 6.18 V_RMS_ and 6.14 V_RMS_, respectively, in DI water and up to 6.14 V_RMS_ and 6.11 V_RMS_, respectively, in the 0.9% NaCl solution, after which point they would reach a maximum temperature of 100 °C.

The remaining devices (devices 1, 3, 5, and 6) have a safe operating voltage of up to a maximum of 5.01 V_RMS_, 5.1 V_RMS_, 4.78 V_RMS_, and 4.90 V_RMS_, respectively, in DI water and up to 4.95 V_RMS_, 5.06 V_RMS_, 4.75 V_RMS_, and 4.82 V_RMS_, respectively, in the 0.9% NaCl solution. 

**Figure 6:**
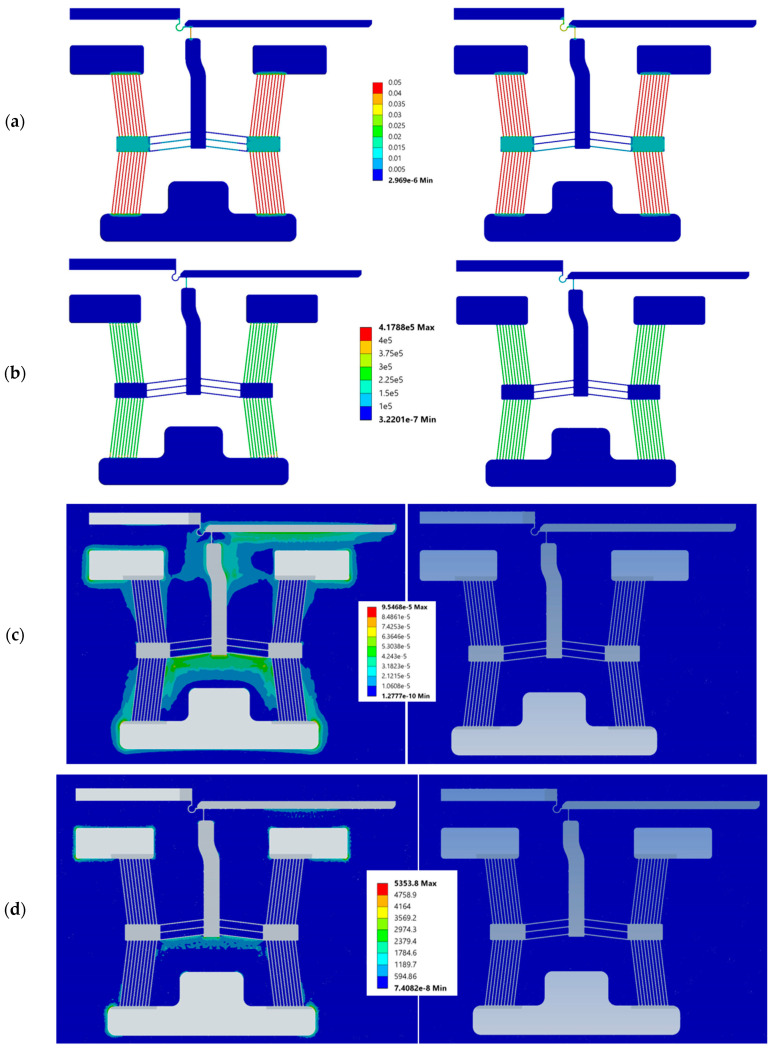
Numerically calculated contours when operating in 0.9% NaCl solution (left) and DI water (right) of (**a**) current density through the device, (**b**) Joule heating in the device domain, (**c**) current density through the fluid, and (**d**) Joule heating in the fluid, at an input voltage of 5 V_RMS_. Legends are as follows: current density in mA/µm^2^, and Joule heating in pW/µm^3^.

Table 4 shows the temperature percentage differences in the devices when operating in DI water versus the 0.9% NaCl solution. Note that the differences are relatively small, with the highest tip temperature differences being exhibited by devices 5 and 6 (3.65% and 3.66%, respectively), and the highest maximum temperature percentage differences of 1.61% and 1.70% exhibited by devices 1 and 6, respectively. These differences are attributed to the difference in electrical resistivity of the test medium. Recall that the electrical resistivity of the 0.9% NaCl solution was measured to be 0.621 Ω·m, while that of the DI water was measured to be 2400 Ω·m. Figure 6 shows the plots of current density and Joule heat of device 1.

With reference to Figure 6, the percentage difference between operation in DI water and the 0.9% NaCl solution becomes apparent. While the current density and respective Joule heat of the device are similar when operating in both media, current flow through the 0.9% NaCl solution is orders of magnitude greater than that in DI water due to its higher electrical conductivity. Joule heating of the fluid is, thus, also greater and, hence, the fluid experiences an increase in temperature. This implies that heat loss by conduction from the device to the surrounding medium shall be greater in the case of DI water, and this explains the temperature differences exhibited by the devices when functioning in both media.

### 5.2. Structural Characterisation

Device tip or end-effector displacement versus RMS voltage were used to characterise the devices’ structural performance in the 0.9% NaCl solution. The experimental readings were compared to those calculated numerically. Recall from Section 1 that, for a MEMS ETA to qualify as a human RBC characterisation tool, it must be able to generate tip displacements similar to the compression that an RBC would experience when travelling through capillaries. That is, the device must produce tip displacement of at least 7 µm in fully submerged conditions. 

#### 5.2.1. Frequency Study

The scope of a frequency independence study is to verify that, when a device is activated using a high-frequency input power source, the device’s structural performance with respect to the RMS voltage of the input signal is similar to that exhibited when supplied with the same DC voltage. Similar to the procedure adopted in [15], a frequency study was performed on all the devices and in air conditions. Here, a 15 kHz sine waveform was utilised. Recall that this study aims to provide confidence that the chosen input source is suitable for the device to perform its intended function prior to submerging it for testing in the desired medium. Note that the reason for opting to use a sine wave in this work rather than a square waveform, as previously used in [15], is that a square pulse becomes highly distorted when operating in higher-conductivity fluids such as the 0.9% NaCl solution, due to the signal source slew-rate limit when driving low-resistance loads. This is not the case for a sine wave, as the latter requires a lower slew-rate value. Also note that the waveform’s profile was verified mathematically prior to publishing any data.

**Figure 7:**
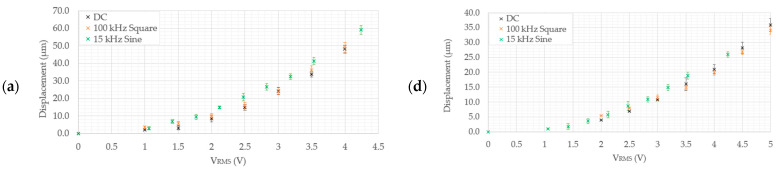

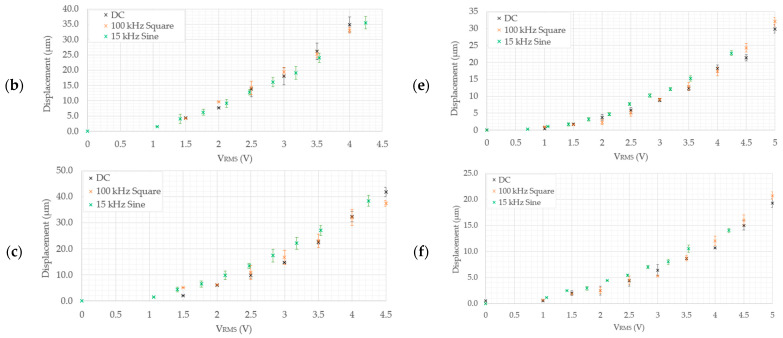
Tip displacement versus RMS voltage graphs of (**a**) device 1, (**b**) device 2, (**c**) device 3, (**d**) device 4, (**e**) device 5, and (**f**) device 6. Note that, in the case of device 5, the displacement data-points are the total change in the gap opening. The displacement versus RMS voltage data of devices 1–4 when subjected to DC and 100 kHz square input sources were extracted from [15].

Figure 7 shows the tip displacements versus the RMS voltage when activated using a DC source, a 100 kHz square waveform, and a 15 kHz sine waveform. With reference to Figure 7, the devices’ performance when subjected to an RMS input is evidently similar to that shown when subjected to a DC source; therefore, this proves that a 15 kHz sine wave is also a suitable activation input for the devices. Note that, due to hardware limitations, a maximum working peak voltage of 5 V, which equates to 4.24 V_RMS_, was possible in this work.

#### 5.2.2. Structural Performance in 0.9% NaCl Solution

Within this subsection, the structural performance of all six devices when fully submerged in the 0.9% NaCl solution is demonstrated numerically and experimentally. Tip or end-effector displacement is, once again, the performance parameter monitored, and a device eligible for underwater biomedical procedures is one that generates displacements larger than 7 µm within the thermally acceptable voltage ranges identified in Section 5.1. Lastly, the performance of the devices in the solution is compared to that in DI water.

**Figure 8:**
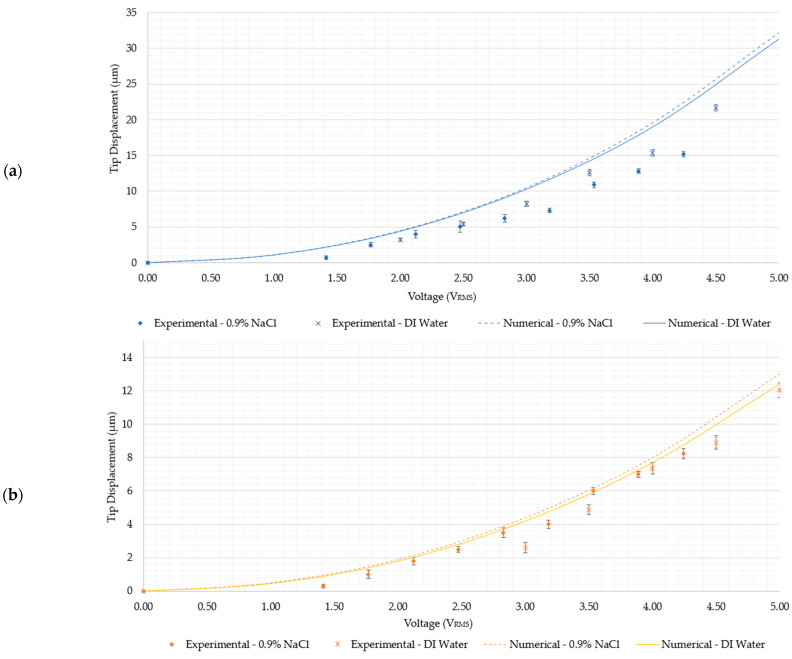

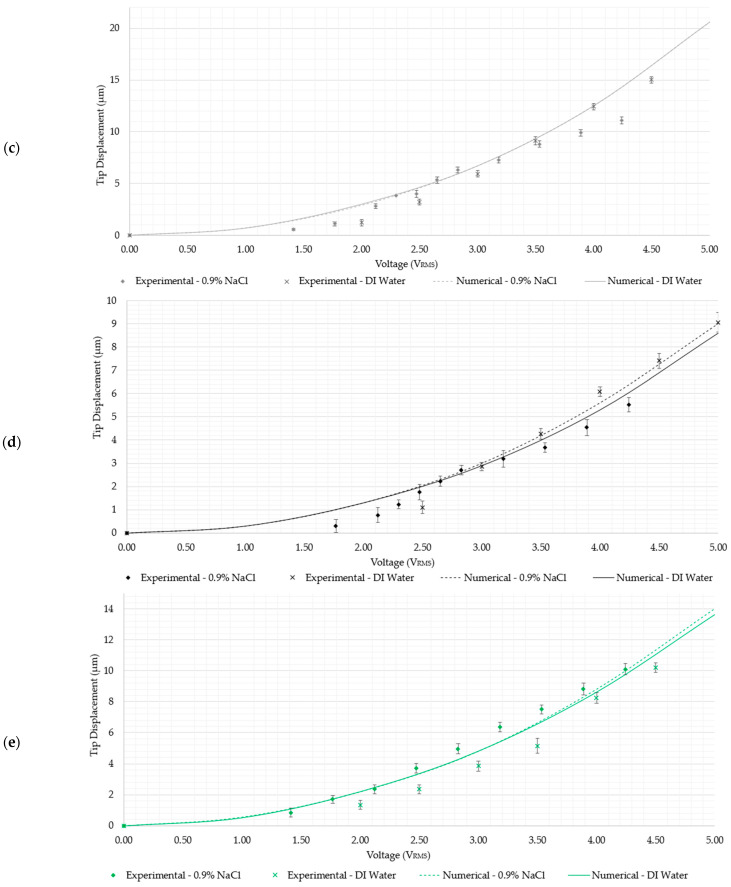

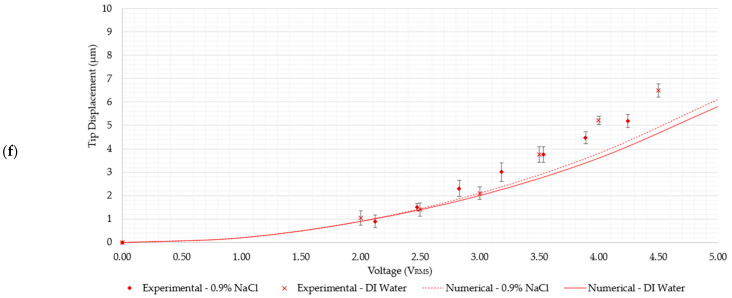
Experimental and numerical tip displacement versus RMS voltage graphs of (**a**) device 1, (**b**) device 2, (**c**) device 3, (**d**) device 4, (**e**) device 5, and (**f**) device 6 in the 0.9% NaCl solution and DI water. The experimental tip displacement versus RMS voltage data of devices 1, 2, and 4 in DI water were extracted from [15]. The numerically calculated tip displacement versus voltage data of devices 3, 5, and 6 in DI water as well as the experimental tip displacement versus voltage data of device 3 in DI water have been reprinted with permission from [19] Sciberras, T.; Mollicone, P.; Demicoli, M.; Grech, I.; Sammut, N.; Mallia, B. “MEMS Electrothermal Actuators for Underwater Manipulation and Mechanical Characterisation of Human Red Blood Cells.”, in *2023 Symposium on Design, Test, Integration & Packaging of MEMS/MOEMS (DTIP)*; 2023; pp 1–4.

Recall from Figure 5 that all the devices besides 2 and 4 may operate up to an input voltage of 4.5 V_RMS_ so as not to reach temperatures of 100 °C. With reference to Figure 5 and Figure 8, the following observations can be outlined:

- Devices 1 and 3 generate the highest tip displacements in both media. The experimental tip displacements of devices 1 and 3 were measured at 21.7 µm and 15 µm, respectively, at an input voltage of 4.5 V_RMS_ in DI water. The numerically calculated tip displacements of devices 1 and 3 in DI water at the same input voltage were 25 µm and 16.6 µm, respectively. The experimental tip displacements of devices 1 and 3 in the 0.9% NaCl solution were measured at 15.18 µm and 11.1 µm at an input voltage of 4.24 V_RMS_. Their numerically calculated counterparts were 22.5 µm for device 1 and 10.9 µm for device 3. This implies that both devices are well-suited for biomedical applications requiring devices to function whilst fully submerged in aqueous solutions up to a maximum input voltage of 4.5 V_RMS_.
- Devices 2 and 4 exhibited tip displacements of 12.0 µm and 9.1 µm, respectively, at an input voltage of 5 V_RMS_ in DI water when measured experimentally. Also experimentally, in the 0.9% NaCl solution, devices 2 and 4 exhibited tip displacements of 8.3 µm and 5.5 µm, respectively, at an input voltage of 4.24 V_RMS_. The equivalent numerically calculated values for device 2 were 12.4 µm and 9.2 µm in DI water and the 0.9% NaCl solution, respectively. Meanwhile, the numerically calculated values of device 4 were 8.6 µm and 6.4 µm in DI water and the 0.9% NaCl solution, respectively. Similar to devices 1 and 3, device 2 is also a potential candidate for human RBC characterisation in aqueous solutions. While device 4 exceeds the 7 µm threshold in DI water, unfortunately, it does not exceed it in the 0.9% NaCl solution with the maximum available voltage supply from the available signal generator. Note, however, that by extrapolating the trend as exhibited in Figure 8e, the device may produce a 7 µm tip displacement with a 5 V_RMS_ input. With reference to Figure 5, devices 2 and 4 may be operated up to 5 V_RMS_ from an electro-thermal standpoint, since they do not reach a maximum temperature of 100 °C.
- As measured experimentally, device 5 exhibits a total gap opening of 10.2 µm at 4.5 V_RMS_ and 10.1 µm at 4.24 V_RMS_ in DI water and the 0.9% NaCl solution, respectively. The corresponding numerically calculated values are 10.1 µm and 11.3 µm in the 0.9% NaCl solution and DI water, respectively. This implies that device 5 is also a suitable candidate for submerged, aqueous biomedical applications.
- Device 6 is the least-performing device, in that it was seen to produce experimental tip displacements of 6.5 µm at 4.5 V_RMS_ and 5.19 µm at 4.24 V_RMS_ in DI water and the 0.9% NaCl solution, respectively. The numerically derived values were 4.3 µm and 4.7 µm in 0.9% NaCl and DI water, respectively. Although marginally below specification, this device needs a design intervention prior to being a potential candidate for underwater biomedical applications. The amplification mechanism is the target element requiring optimisation here, since the electro-thermal driver section has been successfully utilised in all the other devices.

As previously concluded in [15,19], the main differences between numerically calculated and experimentally observed tip displacements are predominantly manufacturing process- or environmentally related. Numerical models are carried out in nominal dimensions, whereby no process-related tolerances are included. Also, no prestress is considered; therefore, any process-related residual stresses are ignored in numerical models. This means that process-induced geometrical tolerances on the physical parts, together with internal residual stresses, contribute towards the difference exhibited in the performance of physical prototypes when compared with numerically calculated results. Also, experimental testing is not carried out in a tightly controlled climatic chamber, which can induce discrepancies. Another contributing factor here is that, in the numerical models, conduction is the only heat transfer mechanism considered. While this seems to be the case for devices 1 to 4, where most experimental readings are lower than the numerically calculated readings, devices 5 and 6 exhibit a good amount of experimental data-points that are higher than the numerically calculated readings. In this case, it is likely that process-related dimensional variations were the predominant cause of the discrepancies witnessed.

## 6. Conclusions

This work made use of devices whose geometrical form and functional characteristics were previously reported in [19,31]. All six devices were fabricated using the commercially available SOIMUMP micromachining process and made use of a cascaded V-shaped electro-thermal driver to perform their intended functions. To recap, devices 2 and 4 have primary V-shaped drivers with five beams per side, while the rest have ten. Devices 1 and 2 have identical amplification mechanisms that differ from those of devices 3 and 4. Devices 3 and 4 have the same amplification mechanisms [31]. Devices 5 and 6 have unique compliant mechanisms, whereby device 5 is a tweezer-type mechanism well-suited for both manipulation and characterisation activities, while device 6 is better suited for compression-only tasks, such as characterisation [19]. 

The electro-thermo-mechanical characterisation of all six devices was performed numerically using a simplified sequential-coupling method, as detailed in [19]. This method is based purely on the finite element method, and heat transfer from the device to the surrounding fluid is only possible by conduction. Here, it was possible to determine safe working voltage ranges for the individual devices so that the resulting Joule heating would not generate overly high temperatures that do not comply with the specifications outlined in Section 1. These specifications include the notion that no region on the device shall reach a maximum temperature of 100 °C and that tip temperatures must not exceed 40 °C in submerged conditions. With this numerical modelling method, it was determined that devices 2 and 4 may function with an input of up to 5 V_RMS_. Despite not having been tested beyond 4.24 V_RMS_, devices 1, 3, 5, and 6 have a safe working voltage of 4.95 V_RMS_, 5.06 V_RMS_, 4.75 V_RMS_, and 4.82 V_RMS_, respectively, in the 0.9% NaCl solution, as calculated numerically. Tip temperatures are of no concern, as seen in Figure 5, since they are all well-below 40 °C. From a mechanical performance perspective, a minimum tip or end-effector displacement of 7 µm at the safe operating voltages identified was set as the specification, since a human RBC is forced to deform by approximately this amount when travelling through capillaries. As was the case in [15], devices 1 and 3 generate the highest displacements, and this is attributed to two main factors. Firstly, when compared with devices 2 and 4, their primary drivers incorporate 10 beams per side rather than 5. The 10 beams are more tightly spaced and, therefore, contain more heat in their vicinity, allowing less temperature loss by conduction and resulting in more thermal expansion and a larger total tip displacement. The main differences in tip displacement between devices 1 and 3 and devices 2 and 4 are then purely attributed to the different amplification beam hinge features. As previously detailed in [15,31], devices 3 and 4 have a stiffer amplification beam hinge configuration compared to devices 1 and 2. Devices 5 and 6 both share the same cascaded V-shaped driver as devices 1 and 3. They are both unique in the way in which they transfer the linear motion from the driver to their end-effectors. Numerically, device 5 poses itself as a good candidate for the characterisation and manipulation of human RBCs in both aqueous solutions. Device 6, however, produces displacements that may not be well-suited for the characterisation of human RBCs, since its numerically calculated tip displacement is below 7 µm.

The structural performance of all six devices was verified experimentally in both DI water and 0.9% NaCl using MEMS-dedicated testing equipment. A frequency study was performed in air prior to submerging the devices in their respective media. In this work, a 15 kHz sine waveform was utilised on all devices, and a 100 kHz square waveform was utilised solely for devices 5 and 6. The frequency study concerning devices 1 to 4 in the presence of the 100 kHz square waveform had been previously reported in [15]. It was determined that the devices’ response to the RMS input of both the higher-frequency square and sine waveform inputs was similar to that of a DC input of an equal magnitude. 

While the experimental data suggest that all but device 6 are potential candidates for the characterisation and manipulation of human RBCs in submerged conditions, devices 1 and 3 are the best-performing devices, since they generate the highest tip displacements in both DI water and the 0.9% NaCl solution. This was attributed to them having 10 beams that are tighter-spaced when compared with devices 2 and 4. Device 4 was also seen as a low performer, since it exhibited a maximum tip displacement of 5.52 µm in the 0.9% NaCl solution. It is worth noting, however, that, due to hardware limitations, 4.24 V_RMS_ was the maximum possible applied input voltage. Device 4, however, could operate at 5 V_RMS_, since its thermal performance at 5 V_RMS_ allowed it, so it may be possible for the 7 µm specification to be achieved. Device 6 was ruled out as a candidate for biomedical procedures based on the specifications listed in Section 1. It may, however, be used for other applications that do not have such strict specifications. Other potential applications include the characterisation of micro-objects that are not temperature-sensitive, with procedures performed in air. Device 6 may also be used as a MEMS electro-thermal switch. For device 6 to be suitable for the specifications detailed in Section 1, geometric optimisation would be required. Lastly, in most instances, the numerically calculated tip displacements are in good agreement with the experimental data. The main differences between the numerically calculated and experimentally observed tip displacements are mainly attributed to geometry or environmental effects. 

## Figures and Tables

**Table 1:** Thermophysical properties of SOI and pad metal layers. Unless otherwise specified, all the values besides the electrical resistivity of SOI were extracted from [32]. The resistivity of SOI was taken from [31]. Designations “x” and “y” refer to in-plane areas whereas “z” refers to an out-of-plane area, and all the properties are assumed to be at a reference temperature of 22 °C. © 2024 IEEE. Reprinted with permission from Sciberras, T.; Demicoli, M.; Grech, I.; Mallia, B.; Mollicone, P.; Sammut, N. “Experimental and Numerical Analysis of MEMS Electrothermal Actuators with Cascaded V-shaped Mechanisms,” *2022 Symposium on Design, Test, Integration and Packaging of MEMS/MOEMS (DTIP)*, Pont-a-Mousson, France, 2022, pp. 1–5.

Property Designation	SOI	Pad Metal (Gold)
Density [g/(cm)^3^]	2.50	19.30
Electrical resistivity, ρ [µ.Ω.m]	125	2.86 × 10^−2^
Coefficient of thermal expansion, α [µm/m.K]	[31,32]	N/A
Thermal conductivity, k [W/m.K]	148	297
Specific heat capacity, c [J/kg.K]	712	128.7
Shear modulus, G [GPa]	G_yz_ = G_zx_ = 79.6, G_xy_ = 50.9	N/A
Poisson’s ratio, ν	ν_yz_ = 0.36, ν_zx_ = 0.29, ν_xy_ = 0.064	0.35
Young’s modulus, E [GPa]	E_x_ = E_y_ = 169, E_z_ = 130	57

**Table 2:** ETA device’s configurations and designations. © 2024 IEEE. Reprinted with permission from [31] Sciberras, T.; Demicoli, M.; Grech, I.; Mallia, B.; Mollicone, P.; Sammut, N. “Experimental and Numerical Analysis of MEMS Electrothermal Actuators with Cascaded V-shaped Mechanisms,” *2022 Symposium on Design, Test, Integration and Packaging of MEMS/MOEMS (DTIP)*, Pont-a-Mousson, France, 2022, pp. 1–5 and [19] Sciberras, T.; Mollicone, P.; Demicoli, M.; Grech, I.; Sammut, N.; Mallia, B. “MEMS Electrothermal Actuators for Underwater Manipulation and Mechanical Characterisation of Human Red Blood Cells.”, in *2023 Symposium on Design, Test, Integration & Packaging of MEMS/MOEMS (DTIP)*; 2023; pp 1–4.

Device Configuration	Reference	Designation in Reference	Designation in Current Text
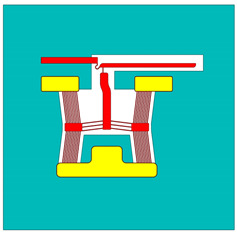	[31]	Device 1	Device 1
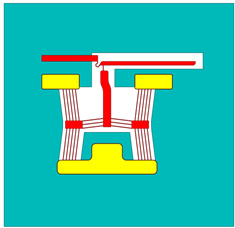		Device 2	Device 2
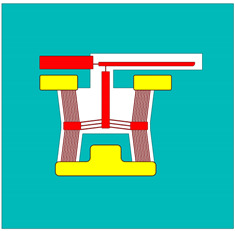		Device 3	Device 3
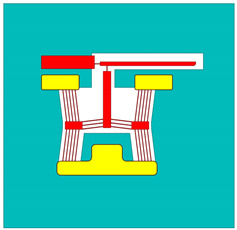		Device 4	Device 4
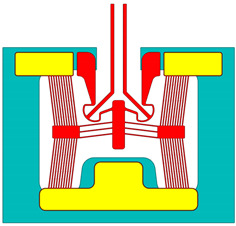	[19]	Device a	Device 5
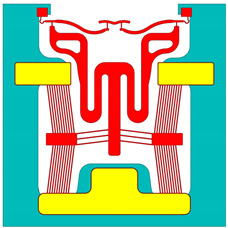		Device b	Device 6

**Table 4:** Temperature percentage difference in DI water against 0.9% NaCl solution.

Voltage (V_RMS_)	Percentage Difference (%)											
	Tip Temperature						Maximum Temperature					
	Device Number						Device Number					
	1	2	3	4	5	6	1	2	3	4	5	6
1	0.10	0.09	0.02	0.08	0.18	0.23	0.25	0.12	0.06	0.07	0.15	0.35
2	0.41	0.38	0.10	0.34	0.71	0.90	0.74	0.39	0.18	0.24	0.43	1.01
3	0.92	0.84	0.22	0.76	1.51	1.93	1.15	0.66	0.28	0.40	0.65	1.55
4	1.62	1.48	0.39	1.33	2.53	3.22	1.43	0.87	0.35	0.52	0.80	1.90
5	2.48	2.29	0.60	2.06	3.65	3.66	1.61	1.01	0.39	0.61	0.89	1.70

## Data Availability

The original contributions presented in the study are included in the article, further inquiries can be directed to the corresponding author.

## Abbreviations

The following abbreviations are used in this manuscript:

BC	Boundary condition
ETA	Electro-thermal actuator
MEMS	Microelectromechanical system
SOI	Silicon-on-insulator
RBC	Red blood cell
DI	Deionised

## References

[1] Califf R.M. (2018). Biomarker Definitions and Their Applications. Exp. Biol. Med..

[2] Takayama Y., Perret G., Kumemura M., Ataka M., Meignan S., Karsten S.L., Fujita H., Collard D., Lagadec C., Tarhan M.C. (2018). Developing a MEMS Device with Built-in Microfluidics for Biophysical Single Cell Characterization. Micromachines.

[3] Nader E., Skinner S., Romana M., Fort R., Lemonne N., Guillot N., Gauthier A., Antoine-Jonville S., Renoux C., Hardy-Dessources M.-D. (2019). Blood Rheology: Key Parameters, Impact on Blood Flow, Role in Sickle Cell Disease and Effects of Exercise. Front. Physiol..

[4] Renoux C., Faivre M., Bessaa A., Da Costa L., Joly P., Gauthier A., Connes P. (2019). Impact of Surface-Area-to-Volume Ratio, Internal Viscosity and Membrane Viscoelasticity on Red Blood Cell Deformability Measured in Isotonic Condition. Sci. Rep..

[5] Parrow N.L., Violet P.-C., Tu H., Nichols J., Pittman C.A., Fitzhugh C., Fleming R.E., Mohandas N., Tisdale J.F., Levine M. (2018). Measuring Deformability and Red Cell Heterogeneity in Blood by Ektacytometry. J. Vis. Exp..

[6] Chen X., Feng L., Jin H., Feng S., Yu Y. (2009). Quantification of the Erythrocyte Deformability Using Atomic Force Microscopy: Correlation Study of the Erythrocyte Deformability with Atomic Force Microscopy and Hemorheology. Clin. Hemorheol. Microcirc..

[7] Fornal M., Lekka M., Pyka-Fościak G., Lebed K., Grodzicki T., Wizner B., Styczeń J. (2006). Erythrocyte Stiffness in Diabetes Mellitus Studied with Atomic Force Microscope. Clin. Hemorheol. Microcirc..

[8] Shakoor A., Gao W., Zhao L., Jiang Z., Sun D. (2022). Advanced Tools and Methods for Single-Cell Surgery. Microsystems Nanoeng..

[9] Da Costa L., Suner L., Galimand J., Bonnel A., Pascreau T., Couque N., Fenneteau O., Mohandas N. (2016). Diagnostic Tool for Red Blood Cell Membrane Disorders: Assessment of a New Generation Ektacytometer. Blood Cells. Mol. Dis..

[10] Bustamante C.J., Chemla Y.R., Liu S., Wang M.D. (2021). Optical Tweezers in Single-Molecule Biophysics. Nat. Rev. Methods Prim..

[11] Musielak M. (2009). Red Blood Cell-Deformability Measurement: Review of Techniques. Clin. Hemorheol. Microcirc..

[12] Cauchi M., Grech I., Mallia B., Mollicone P., Sammut N. (2018). The Effects of Structure Thickness, Air Gap Thickness and Silicon Type on the Performance of a Horizontal Electrothermal MEMS Microgripper. Actuators.

[13] Kim K., Cheng J., Liu Q., Wu X.Y., Sun Y. (2010). Investigation of Mechanical Properties of Soft Hydrogel Microcapsules in Relation to Protein Delivery Using a MEMS Force Sensor. J. Biomed. Mater. Res. A.

[14] Yallew T.S., Pantano M.F., Bagolini A. Design and Finite Element Analysis of an Electrothermally Actuated Microgripper for Biomedical Applications. Proceedings of the 2021 Symposium on Design, Test, Integration & Packaging of MEMS and MOEMS (DTIP).

[15] Sciberras T., Demicoli M., Grech I., Mallia B., Mollicone P., Sammut N. (2023). Thermo-Mechanical Fluid–Structure Interaction Numerical Modelling and Experimental Validation of MEMS Electrothermal Actuators for Aqueous Biomedical Applications. Micromachines.

[16] Nemani K.V., Moodie K.L., Brennick J.B., Su A., Gimi B. (2013). In Vitro and in Vivo Evaluation of SU-8 Biocompatibility. Mater. Sci. Eng. C. Mater. Biol. Appl..

[17] Potrich C., Lunelli L., Bagolini A., Bellutti P., Pederzolli C., Verotti M., Belfiore N.P. (2018). Innovative Silicon Microgrippers for Biomedical Applications: Design, Mechanical Simulation and Evaluation of Protein Fouling. Actuators.

[18] Joshitha C., Ragavyshnavi D., Priyanka D.S., Nagarjuna P., Sreeja B.S. (2021). Electrothermal Micro Tweezer for Biomedical Applications. {IOP} Conf. Ser. Mater. Sci. Eng..

[19] Sciberras T., Mollicone P., Demicoli M., Grech I., Sammut N., Mallia B. MEMS Electrothermal Actuators for Underwater Manipulation and Mechanical Characterisation of Human Red Blood Cells. Proceedings of the 2023 Symposium on Design, Test, Integration & Packaging of MEMS/MOEMS (DTIP).

[20] Singh M., Stoltz J. (2002). Influence of Temperature Variation from 5 Degrees C to 37 Degrees C on Aggregation and Deformability of Erythrocytes. Clin. Hemorheol. Microcirc..

[21] Xu Z., Zheng Y., Wang X., Shehata N., Wang C., Sun Y. (2018). Stiffness Increase of Red Blood Cells during Storage. Microsystems Nanoeng..

[22] Matrai A.A., Varga G., Tanczos B., Barath B., Varga A., Horvath L., Bereczky Z., Deak A., Nemeth N. (2021). In Vitro Effects of Temperature on Red Blood Cell Deformability and Membrane Stability in Human and Various Vertebrate Species. Clin. Hemorheol. Microcirc..

[23] Lecklin T., Egginton S., Nash G.B. (1996). Effect of Temperature on the Resistance of Individual Red Blood Cells to Flow through Capillary-Sized Apertures. Pflugers Arch..

[24] Fildes J., Fisher S., Sheaff C.M., Barrett J.A. (1998). Effects of Short Heat Exposure on Human Red and White Blood Cells. J. Trauma Acute Care Surg..

[25] Potter R.F., Groom A.C. (1983). Capillary Diameter and Geometry in Cardiac and Skeletal Muscle Studied by Means of Corrosion Casts. Microvasc. Res..

[26] Attaway J. Osmosis and Tonicity. https://www.khanacademy.org/science/biology/membranes-and-transport/diffusion-and-osmosis/a/osmosis.

[27] Lukose J., Shastry S., Mithun N., Mohan G., Ahmed A., Chidangil S. (2020). Red Blood Cells under Varying Extracellular Tonicity Conditions: An Optical Tweezers Combined with Micro-Raman Study. Biomed. Phys. Eng. Express.

[28] Moritz M.L. (2019). Why 0.9% Saline Is Isotonic: Understanding the Aqueous Phase of Plasma and the Difference between Osmolarity and Osmolality. Pediatr. Nephrol..

[29] Sameoto D., Hubbard T., Kujath M. (2004). Operation of Electrothermal and Electrostatic MUMPs Microactuators Underwater. J. Micromechanics Microengineering.

[30] Mukundan V., Pruitt B. (2009). MEMS Electrostatic Actuation in Conducting Biological Media. Microelectromech. Syst. J..

[31] Sciberras T., Demicoli M., Grech I., Mallia B., Mollicone P., Sammut N. Experimental and Numerical Analysis of MEMS Electrothermal Actuators with Cascaded V-Shaped Mechanisms. Proceedings of the 2022 Symposium on Design, Test, Integration & Packaging of MEMS/MOEMS.

[32] Cowen A., Hames G., Monk D., Wilcenski S., Hardy B. (2011). SOIMUMPs Design Handbook.

[33] Hickey R., Sameoto D., Hubbard T., Kujath M. (2002). Time and Frequency Response of Two-Arm Micromachined Thermal Actuators. J. Micromechanics Microengineering.

[34] Qasem N., Generous M.M., Qureshi B., Zubair S. (2021). A Comprehensive Review of Saline Water Correlations and Data: Part II—Thermophysical Properties. Arab. J. Sci. Eng..

[35] ANSYS (2013). Academic Research Mechanical, Release 15.0, Help System, ANSYS Fluent User’s Guide.

[36] Generous M.M., Qasem N., Qureshi B., Zubair S. (2020). A Comprehensive Review of Saline Water Correlations and Data-Part I: Thermodynamic Properties. Arab. J. Sci. Eng..

